# Occult small bowel perforation presenting as extensive subcutaneous emphysema without skin breach

**DOI:** 10.1093/jscr/rjag606

**Published:** 2026-07-21

**Authors:** Amanda Barden, Utsah Bhattacharya, Anang Pangeni, Gandrasupalli Harinath, Prashant Naik

**Affiliations:** General Surgery Department, East Kent Hospitals University NHS Foundation Trust, Ashford TN24 0LZ, United Kingdom; General Surgery Department, East Kent Hospitals University NHS Foundation Trust, Ashford TN24 0LZ, United Kingdom; General Surgery Department, East Kent Hospitals University NHS Foundation Trust, Ashford TN24 0LZ, United Kingdom; General Surgery Department, East Kent Hospitals University NHS Foundation Trust, Ashford TN24 0LZ, United Kingdom; General Surgery Department, East Kent Hospitals University NHS Foundation Trust, Ashford TN24 0LZ, United Kingdom

**Keywords:** subcutaneous emphysema, small bowel perforation, entero-subcutaneous tract, enterocutaneous fistula, IPAA

## Abstract

A woman with ulcerative colitis status post total pan-proctocolectomy and ileal pouch-anal anastomosis 17 years prior presented with recurrent abdominal pain, vomiting, and weight loss. Following multiple admissions being managed for subacute bowel obstruction and anastomotic narrowing without functional obstruction, she was found to have extensive subcutaneous emphysema from the right inguinal canal to the thorax on imaging. The aetiology was fistulation between a small bowel loop and the anterior abdominal wall without any skin breach. Management involved drainage and control of sepsis with formation of a controlled enterocutaneous fistula to avoid a laparotomy in a nutritionally compromised patient. This case highlights that recurrent obstructive symptoms in patients with complex surgical histories may have evolving intra-abdominal pathology, including occult perforation and fistulation. In selected patients, controlled enterocutaneous fistula formation may offer an alternative to laparotomy.

## Introduction

Patients with ulcerative colitis treated with ileal pouch-anal anastomosis (IPAA) often face long-term complications such as pouchitis, strictures, and mechanical obstructions [1]. While fistulation is a known complication [1, 2] (though rare and more common in Crohn’s disease [2]), subcutaneous emphysema arising from enteric fistulation without an external skin opening is rare. In general surgery, subcutaneous emphysema is typically associated with thoracic pathology (e.g. pneumothorax, chest drain insertion, or necrotizing soft tissue infections) [3, 4]. Awareness of such atypical presentations is crucial for timely diagnosis and management.

## Case report

A lady in her 60s presented with a 2-week history of right-sided abdominal pain, intermittent vomiting, and weight loss. She had undergone a total pan-proctocolectomy with IPAA formation 17 years prior for ulcerative colitis but had not been followed up regularly for the last 7 years.

Examination revealed a soft abdomen with marked right iliac fossa tenderness and guarding. Digital rectal examination revealed anal stenosis with a capacious pouch, and computed tomography (CT) demonstrated dilated fluid-filled small bowel loops with a transition point at the anastomosis. She was managed conservatively for suspected subacute obstruction. Outpatient pouchoscopy showed anastomotic narrowing without functional obstruction, and the pouch mucosa appeared normal, excluding pouchitis.

On re-presentation, there was progression of small bowel dilation and thickening of loops in the right abdomen, and she was again managed conservatively for subacute obstruction.

Further deterioration with worsening pain, vomiting, and peritonism led to re-presentation within one month, with elevated inflammatory markers and erythema at the previous ileostomy reversal site leading to admission.

A CT scan showed subcutaneous emphysema tracking from the right inguinal canal to the thorax, with no skin breach (Figs 1 and 2). This appeared to be secondary to fistulation between a thickened small bowel loop within the right abdomen and the anterior abdominal wall, passing via the rectus muscle; however, the exact transition point could not be found. This caused free air to dissect into subcutaneous tissues with no intra-abdominal free fluid or free gas. Small bowel loops were dilated though there were faeces distally, suggesting subacute obstruction (Fig. 2). This was believed to have formed rapidly between her previous and current admission as previous imaging showed no collection, extraluminal gas, or tract formation.

**Figure 1:**
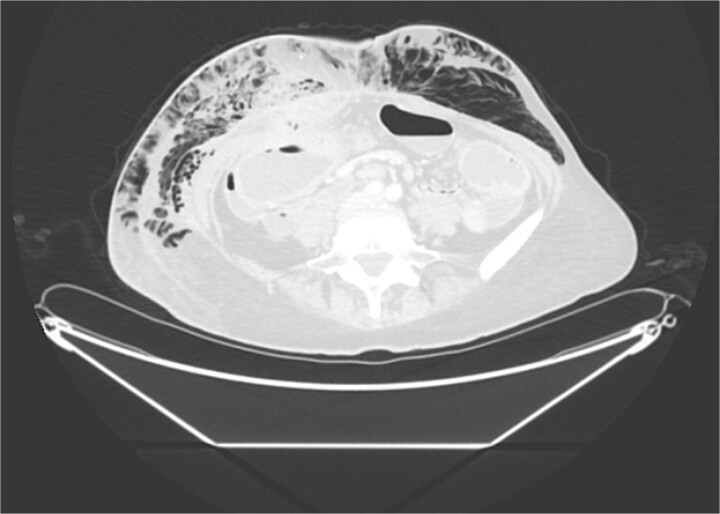
Axial CT demonstrating extensive subcutaneous emphysema within the abdominal wall.

**Figure 2:**
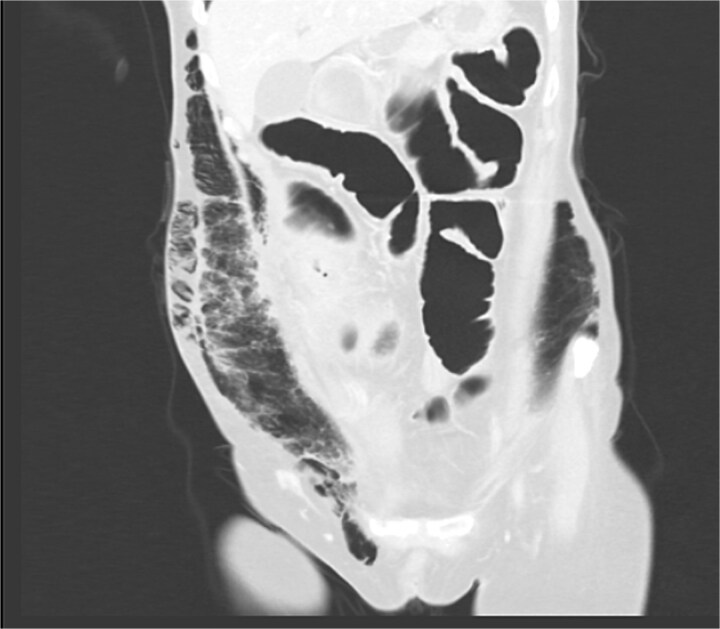
Coronal CT showing tracking of subcutaneous emphysema from the inguinal region to the thoracic wall.

After multidisciplinary discussion, two options were considered: laparotomy with bowel resection and stoma formation, or drainage of the subcutaneous collection to control sepsis and establish a controlled enterocutaneous fistula. Given the patient’s nutritional depletion, the absence of generalized peritonitis, and the possibility of a contained process, limited incisional drainage and debridement were performed. This procedure effectively converted an entero-subcutaneous tract into an enterocutaneous fistula and avoided a laparotomy in a nutritionally depleted patient.

Surgical findings were necrotic tissue with feculent contamination in the adipose layer of anterior abdominal wall, most prominent beneath the previous stoma scar; extensive gas was present within the subcutaneous tissues. For the generation of the enterocutaneous fistula, an incision was made at stoma site scar, deepening of the cavity to find the feculent area, suction and lavage with 1.5 L of saline, debridement of unhealthy fat, and betadine swab wash. The stoma bag was applied to this generated fistula with 1175 ml of fluid drained over 4 days post procedure.

Microbiology cultures grew *Escherichia coli, Enterococcus faecium*, Klebsiella species, and anaerobes. Antibiotic therapy, in liaison with Microbiologists, was tailored to metronidazole, co-trimoxazole, and vancomycin.

These interventions resulted in resolution of subcutaneous emphysema and formation of a controlled enterocutaneous fistula. The patient had an uneventful postoperative recovery and was discharged on Day 10 postoperatively.

## Discussion

This case describes a rare presentation of subcutaneous emphysema secondary to enteric fistulation without skin breach (an entero-subcutaneous tract), occurring in the context of recurrent obstructive symptoms in a patient with previous complex abdominal surgery. Subcutaneous emphysema is typically a thoracic phenomenon [3, 4], and its occurrence secondary to an intra-abdominal enteric fistula is exceptional [5–7].

The exact pathophysiology remains unclear but likely involves a combination of chronic inflammation, increased intraluminal pressure, and localized abscess formation eroding into the abdominal wall causing enteric fistulations; the influence of IPAA on this process remains uncertain.

Cross-sectional imaging was essential in this case, as plain radiography would not have identified the underlying fistulous tract or source of gas.

The absence of pneumoperitoneum in this case may be explained by containment of the perforation within adjacent tissues, preventing free intraperitoneal air. Gas likely dissected along fascial planes via a fistulous tract into the abdominal wall, allowing propagation into subcutaneous tissues resulting in an entero-subcutaneous tract. This mechanism has been described in rare cases of contained perforation, where pressure gradients favour extra-peritoneal gas tracking rather than intraperitoneal free air [3, 5–7].

Management involved the resolution of emphysema and contamination by the generation of an entero-cutaneous tract and targeted antibiotic administration. In contrast to standard management of perforation requiring laparotomy, this case demonstrates that in selected stable patients without generalized peritonitis, particularly in patients who are poor surgical candidates, a more conservative surgical approach may be appropriate and lead to earlier discharge.

In conclusion, recurrent obstructive symptoms in patients with complex surgical histories should prompt consideration of evolving intra-abdominal pathology. Atypical presentations such as subcutaneous emphysema may indicate occult intra-abdominal perforation and fistulation and should prompt early cross-sectional imaging. In selected patients, controlled fistula formation may provide effective management while avoiding laparotomy.
